# Why does it take two to tango? Lifetime fitness consequences of parental care in a burying beetle

**DOI:** 10.1371/journal.pone.0186466

**Published:** 2017-10-31

**Authors:** Ashlee N. Smith, J. Curtis Creighton, Mark C. Belk

**Affiliations:** 1 Biology Department, Brigham Young University, Provo, Utah, United States of America; 2 Department of Biological Sciences, Purdue University Northwest, Hammond, IN, United States of America; University of Missouri Columbia, UNITED STATES

## Abstract

In species that require parental care, each parent can either care for their offspring or leave them in the care of the other parent. For each parent this creates three possible parental care strategies: biparental care, uniparental (male or female) care, and uniparental desertion by either the male or female. The burying beetle, *Nicrophorus orbicollis*, typically exhibits biparental care of offspring, and thus provides a unique system that allows us to compare the fitness benefits of these parental care strategies in an unconfounded way. In this study, we assess the lifetime fitness of biparental care, uniparental care, and uniparental desertion strategies in both male and female *N*. *orbicollis*. Specifically, we tested for increased fitness of the biparental care strategy compared to uniparental care strategies. Second, we test for equality of fitness between uniparental care and uniparental desertion strategies. Surprisingly, biparental care yields lower lifetime fitness for both parents compared to the other two strategies. Also, uniparental care and uniparental desertion strategies yielded equal fitness. The evolution of biparental care in this system is not consistent with the expectation of a mutual fitness benefit. We discuss other potential explanations for the evolution of biparental care in this system.

## Introduction

In species with parental care, each parent has the option to stay with and care for, or to abandon their offspring to the care of the other parent. This creates three potential reproductive strategies: biparental care, uniparental (male or female) care, and leaving the other parent to care for offspring (i.e., uniparental desertion). These three parental care strategies yield two simple predictions about the evolution of parental care behaviors. First, for biparental care to evolve, the fitness of individuals that exhibit biparental behaviors must be greater than individuals that exhibit uniparental care behaviors. Second, for uniparental care to evolve, the mean fitness of the care-giving parents must be equal to the mean fitness of the parents that do not provide parental care (assuming an equal sex ratio) [1–2]. Costs and benefits of biparental and uniparental care have been studied most extensively in birds (See [3] for a review), but almost all of these studies have at least one of the following three confounding conditions: (1) both biparental care and uniparental care are not within the normal range of reproductive behaviors for the species; (2) there is unequal or unknown resource availability for both parents; or (3) a single reproductive bout may not represent lifetime behavior and fitness. Clearly, these conditions potentially confound individual estimates of the costs and benefits of parental care [4].

The burying beetle, *Nicrophorus orbicollis*, is an ideal system for studying evolution of parental care because we can directly address each of the difficulties outlined above. First, although they are typically biparental, each parent is capable of rearing offspring alone [5–8]. The facultative nature of biparental care in this species of burying beetle provides opportunity to experimentally assess fitness of parental care strategies within the normal range of behaviors for the species. Typically, both parents bury and preserve the carcass and feed and protect larvae until they disperse, and both parents regulate brood size through filial cannibalism [9]. Although males generally abandon the brood before the female, they usually remain with the female and the larvae until the larvae reach the third instar stage of development [6, 8, 10–11]. Second, the costs and benefits of biparental and uniparental reproductive strategies can be quantified relative to a known resource amount. *Nicrophorus orbicollis* breed on a small vertebrate carcass, which is the sole food source for both parents and their altricial larvae during the reproductive bout. Because resource availability is fixed and known, we can accurately estimate fitness of parental care strategies and corresponding patterns of energy allocation [4]. Third, we can quantify lifetime reproductive strategies and consequent fitness because the entire reproductive cycle can be completed under laboratory conditions. Previous studies on burying beetles have quantified reproductive output in uniparental and biparental conditions [5–6, 10, 12–14], but have not completely tested fitness of differing parental care strategies.

In this study, we use the burying beetle, *Nicrophorus orbicollis* to evaluate the lifetime fitness consequences of biparental care, uniparental care and uniparental desertion strategies. Parental care is required for the survival of the young, but all three parental strategies are viable options for *N*. *orbicollis* parents. We directly test the prediction that biparental care results in higher fitness than uniparental strategies. In addition, we test the equality of fitness prediction in uniparental care between parents that stay with the brood and parents that leave. To assess fitness benefits of biparental and uniparental care strategies, we compare lifespan, total number of offspring, total offspring mass, mean mass of offspring, number of reproductive bouts, and mean number of offspring per bout in both sexes across all three parental care strategies.

## Materials and methods

### Source ofburying beetles

Adult *N*. *orbicollis* used to generate the laboratory population were captured at the same site in central Wisconsin during summers from 2008 to 2011 using pitfall traps baited with aged chicken. Adult beetles used to generate the laboratory population were captured on privately owned land with the consent of the landowners. We did not collect or use endangered or protected species for these experiments. Wild-caught pairs were placed on a 30g mouse carcass and allowed to breed to generate the laboratory population. The date of eclosion was recorded for all first generation laboratory-bred beetles and was designated as the first day of life. Individuals were placed in small plastic containers (15.6 x 11.6 x 6.7 cm) with *ad libitum* raw chicken liver and maintained on a 14:10 h light:dark cycle.

### Experimental design

To determine costs and benefits of parental care, we quantified reproduction over the lifetime of *N*. *orbicollis* individuals. There were three treatments: biparental care, uniparental care, and uniparental desertion. For the biparental care treatment, both parents were left in the chamber until they dispersed from the brood. For the uniparental care treatment, the non-focal parent was removed within 24 hours of the offspring appearing on the carcass and the focal parent cared for the offspring alone. For the uniparental desertion treatment, the focal parent was removed within 24 hours of the offspring appearing on the carcass and the non-focal parent cared for offspring alone. Thus, in all treatments there was only one parent per trial who was designated as the focal individual and monitored throughout their lifetime for fitness measures. Within each treatment, trials were run for both males and females separately as focal individuals. Accordingly, both males and females (as focal individuals) could be measured for lifetime fitness responses independent of the other parent, and sex as a categorical factor could be included in the analysis. This experimental design resulted in a fully-crossed factorial design with two main effects–parental care treatments (three levels: biparental care, uniparental care, and uniparental desertion) and sex (two levels: males and females)–resulting in six total treatment combinations. All focal beetles reproduced continuously from sexual maturity until death, and were always assigned to the same treatment. Technically, the designation of uniparental care and uniparental desertion treatments applied only to post-hatching parental care. All trials were run concurrently, and all treatments were independent of the others.

#### Biparental care treatment

We began each trial by randomly choosing a genetically unrelated, virgin male and female beetle aged 28–35 days old. One of the pair was designated as the focal individual, and one as the non-focal individual. The focal individual would be followed throughout their lifetime; whereas, the non-focal individual was different for each reproductive bout. The mass, pronotum width, and date of eclosion were recorded for each individual. The pair was placed in a small brood container (16.5 x 15 x 9cm) filled with 6cm of moist soil and given a 30g (± 1.0g) mouse carcass. The containers were kept in an environmental chamber at 21°C on a 14:10 h light:dark cycle.

The brood containers were checked daily, and after larvae arrived on the carcass, the lid of the small brood container was removed and the container was placed in an abandonment chamber (37.5x25.5x14.5cm) flush with an elevated, Styrofoam platform. Two cups (diameter: 8-cm, height: 9.5-cm) were placed in diagonal corners of the abandonment chamber, again, flush with the elevated ledge (see [8]). A thin layer of moist dirt was spread on the ledge and 2cm of moist dirt was placed in each of the cups. Four to five moist paper towels were placed on top of the dirt in the small brood container to maintain moisture. This construction allowed the beetles to move freely in and out of the smaller container.

Abandonment chambers were checked daily and the number of larvae was recorded. The cups in each corner of the abandonment chambers were also checked daily to see if an adult had abandoned the brood. If a parent was found in a cup, its mass and the date were recorded, and it was placed back in the small container with the brood. If a parent abandoned the brood for a second time, it was removed and placed on *ad libitum* chicken liver. The number of offspring present on the carcass did not change between the first and second abandonments by either parent.

When the larvae dispersed into the soil, the remaining parent(s) were removed and weighed and focal beetle was placed on *ad libitum* chicken liver, then placed on a new carcass of the same size two days later with a new genetically unrelated, non-focal, virgin individual. The cycle continued for each focal parent until death. The larvae from each brood reached eclosion 4–5 weeks after dispersal. Number of newly-eclosed adult offspring was used to determine total number of offspring. Each newly-eclosed adult offspring was weighed, and their mass was used to calculate the total offspring mass produced and mean individual offspring mass. Twelve replicates each were completed for both males and females in the biparental care treatment.

#### Uniparental care treatment

This treatment began in the same way as the biparental treatment. However, during each reproductive bout, after larvae arrived on the carcass, the non-focal parent was removed and the focal parent was left to care for the offspring alone. The rest of the experimental trial was carried out in the same way as the biparental care treatment. After the focal parent either abandoned the brood twice or the offspring dispersed, that parent was placed in a container with *ad libitum* chicken liver, then set up to reproduce again two days later with new genetically unrelated, non-focal, virgin beetle. This cycle continued until the focal beetle’s death. Fifteen replicates were completed for females providing uniparental care and twelve replicates were completed for males providing uniparental care.

#### Uniparental desertion treatment

This treatment began the same way as the previous two treatments. However, for each reproductive bout after larvae arrived on the carcass, the focal parent was removed, placed in a container with *ad libitum* chicken liver, and set up to reproduce again two days later with a new genetically unrelated, non-focal, virgin beetle. This cycle continued until the focal beetle’s death. The non-focal parent was left in the container with the brood, and the rest of the experiment was carried out in the same way as the previous two experiments. Fitness determined as number of offspring or offspring mass was measured from the output of each of the reproductive bouts and attributed to the deserting individual, even though the non-focal individual raised them. Twelve replicates each were completed for both deserting males and females.

### Statistical analyses

To determine differences in lifetime fitness among biparental care, uniparental care, and desertion strategies and between males and females, we used six response variables: lifespan, lifetime number of offspring, lifetime total offspring mass, mean individual offspring mass, lifetime number of reproductive bouts, and mean number of offspring per brood. Technically, only lifetime number of offspring represents fitness of the parents. However, inclusion of some measure of offspring quality, offspring size in this case, is important when quality affects offspring survival. Fewer, larger offspring can provide equal fitness to more, smaller offspring when size is linked to survival or reproductive success. We included lifetime total offspring mass and mean individual offspring mass to allow a full exploration of this fitness tradeoff. In addition, lifetime fitness measured as total number of offspring can be influenced by clutch size and reproductive longevity. Lifespan and number of reproductive bouts were included as response variables to assess the effect of parental care strategies on costs of reproduction and patterns of senescence [4]. Finally, in wild populations, number of reproductive bouts may be more limited than in the laboratory populations we used for these experiments. Including the mean number of offspring per brood allowed us to compare how lifetime fitness was accrued, and how fitness might differ if only one or two reproductive bouts were realized under natural conditions. For the first five response variables, the model represented a fully crossed factorial design with two fixed factors: parental type (3 levels, biparental care, uniparental care, and uniparental desertion) and sex (2 levels), and we included the two-way interaction (Proc GLM and Proc GENMOD in SAS; SAS 9.3 SAS Institute, Cary, North Carolina, USA). We evaluated the response variables for normality of residuals and equal variances across treatment combinations. The response variables met these assumptions. We used 95% confidence intervals for post-hoc means comparisons. If the 95% confidence interval of the mean for a given treatment combination did not overlap the mean of another treatment combination, then the two means were considered significantly different.

To determine differences in number of offspring per reproductive attempt among biparental care, uniparental care, and uniparental desertion strategies, we used 2 fixed factors: parental type (3 levels, biparental care, uniparental care, and desertion) and sex (2 levels), and we used number of the reproductive attempt (as an index of time) as a repeated measure. We included all two- and three-way interactions in the analysis. The data were analyzed using PROC GLIMMIX in SAS (SAS 9.3 SAS Institute, Cary, North Carolina, USA).

The goal of this study was to compare the fitness and reproductive patterns of parents that provided biparental care to those that provided uniparental care, and to compare uniparental care to uniparental desertion strategies in metrics common to both sexes. The male uniparental data analyzed in the current paper was previously published by Smith et al. [15], but it was collected at the same time as the uniparental female and biparental male and female data described here.

## Results

**Fig 1:**
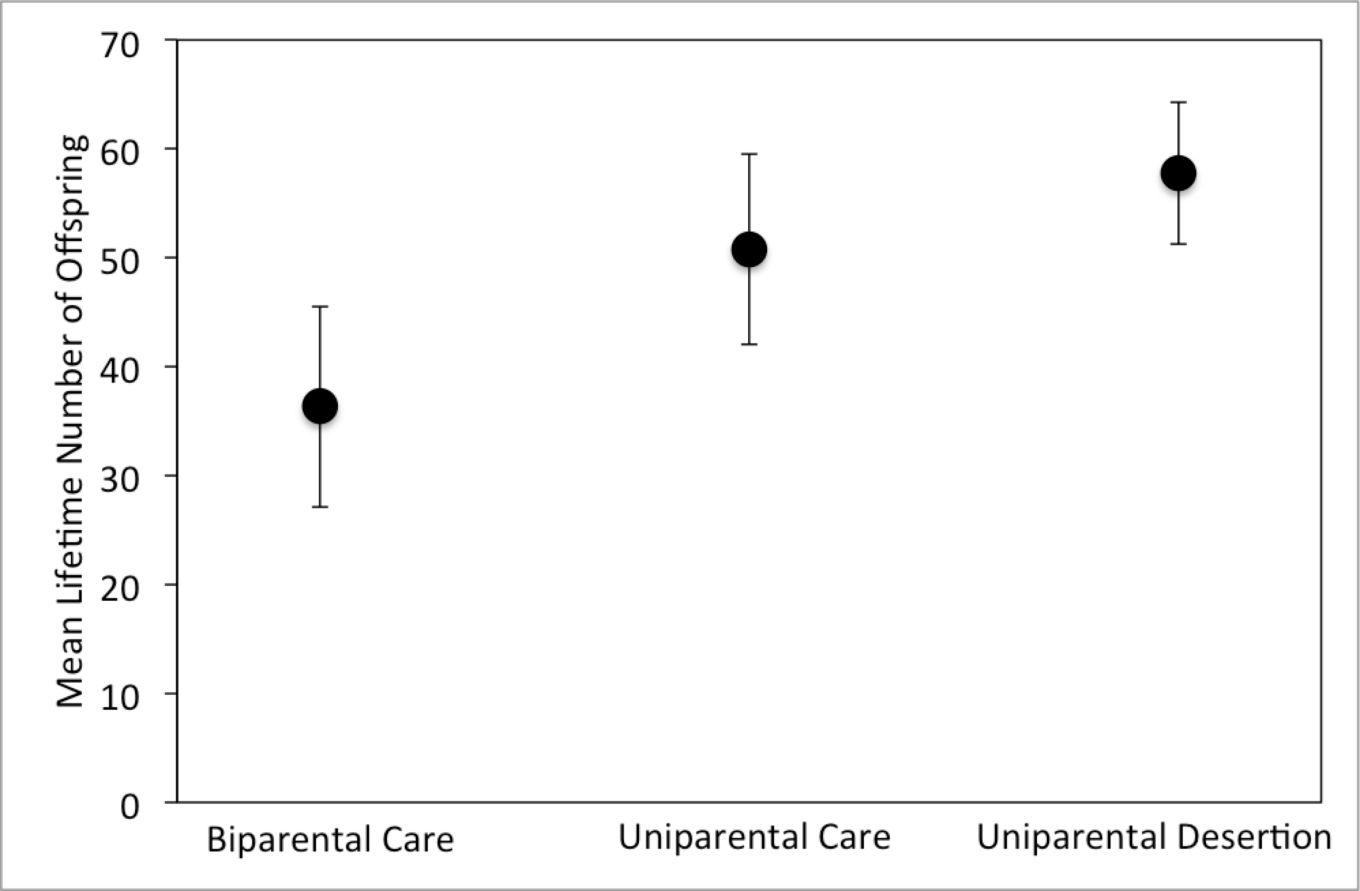
Mean (+/- 95% confidence interval) lifetime number of offspring produced by males and females combined in all three experimental treatments.

Lifetime number of offspring differed significantly among parental types, but sex and the interaction between parental type and sex were not significant (Table 1). Biparental care resulted in significantly fewer offspring than either uniparental care (p = 0.0260) or uniparental desertion (p = 0.0003) strategies, but number of offspring did not differ significantly between uniparental care and uniparental desertion. Biparental care resulted in about 14 fewer offspring over a lifetime (a 28% reduction) compared to uniparental care and about 21 fewer offspring over a lifetime (a 37% reduction) compared to the uniparental desertion strategy (Fig 1).

**Table 1:** Analysis of variance (ANOVA) table for lifetime number of offspring, total offspring mass, individual offspring mass, and lifespan.

Response Variable	Source	Num df/ Den df	F-value	p-value
Lifetime Number of Offspring	Sex	1/98	1.16	0.2846
	Parental Type	2/98	7.13	**0.0013**
	Parental Type*Sex	2/98	0.62	0.5392
Total Offspring Mass	Sex	1/98	3.53	0.0636
	Parental Type	2/98	5.06	**0.0082**
	Parental Type*Sex	2/98	0.60	0.5521
Individual Offspring Mass	Sex	1/98	2.08	0.1525
	Parental Type	2/98	7.16	**0.0013**
	Parental Type*Sex	2/98	7.02	**0.0015**
Lifespan	Sex	1/98	2.18	0.1434
	Parental Type	2/98	3.94	**0.0227**
	Parental Type*Sex	2/98	1.51	0.2256

Significant values are shown in bold.

**Fig 2:**
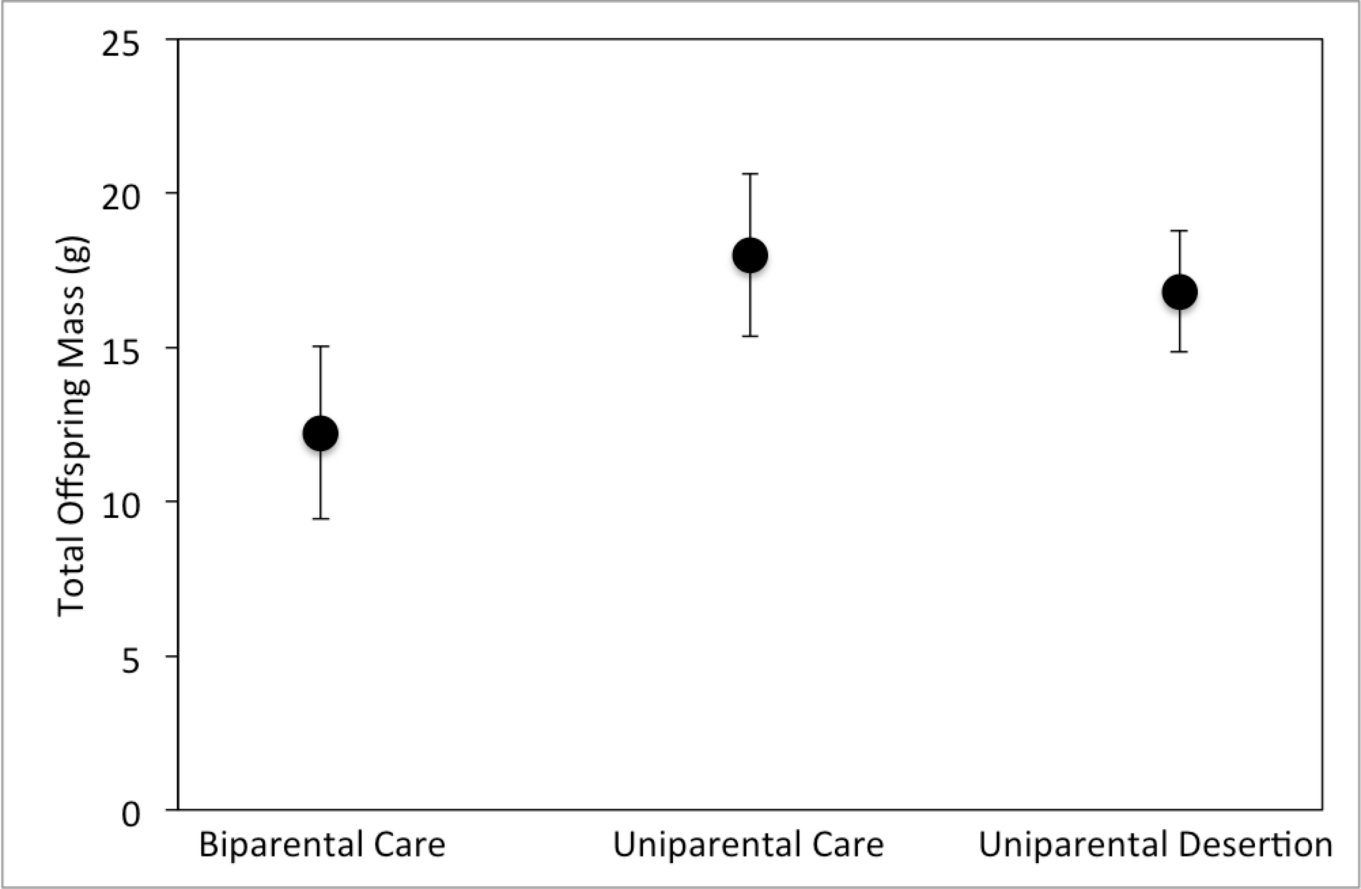
Mean (+/- 95% confidence interval) total offspring mass produced by males and females combined in all three experimental treatments.

Total offspring mass produced differed significantly among parental types, but sex and the interaction between parental type and sex were not significant (Table 1). Biparental care resulted in significantly lower lifetime offspring mass than either uniparental care (p = 0.0037) or uniparental desertion (p = 0.0092) strategies, but lifetime offspring mass did not differ significantly between uniparental care and deserters. Biparental care of offspring produced 32% less total offspring mass compared to uniparental care and 27% less total offspring mass compared to the desertion strategy (Fig 2).

**Fig 3:**
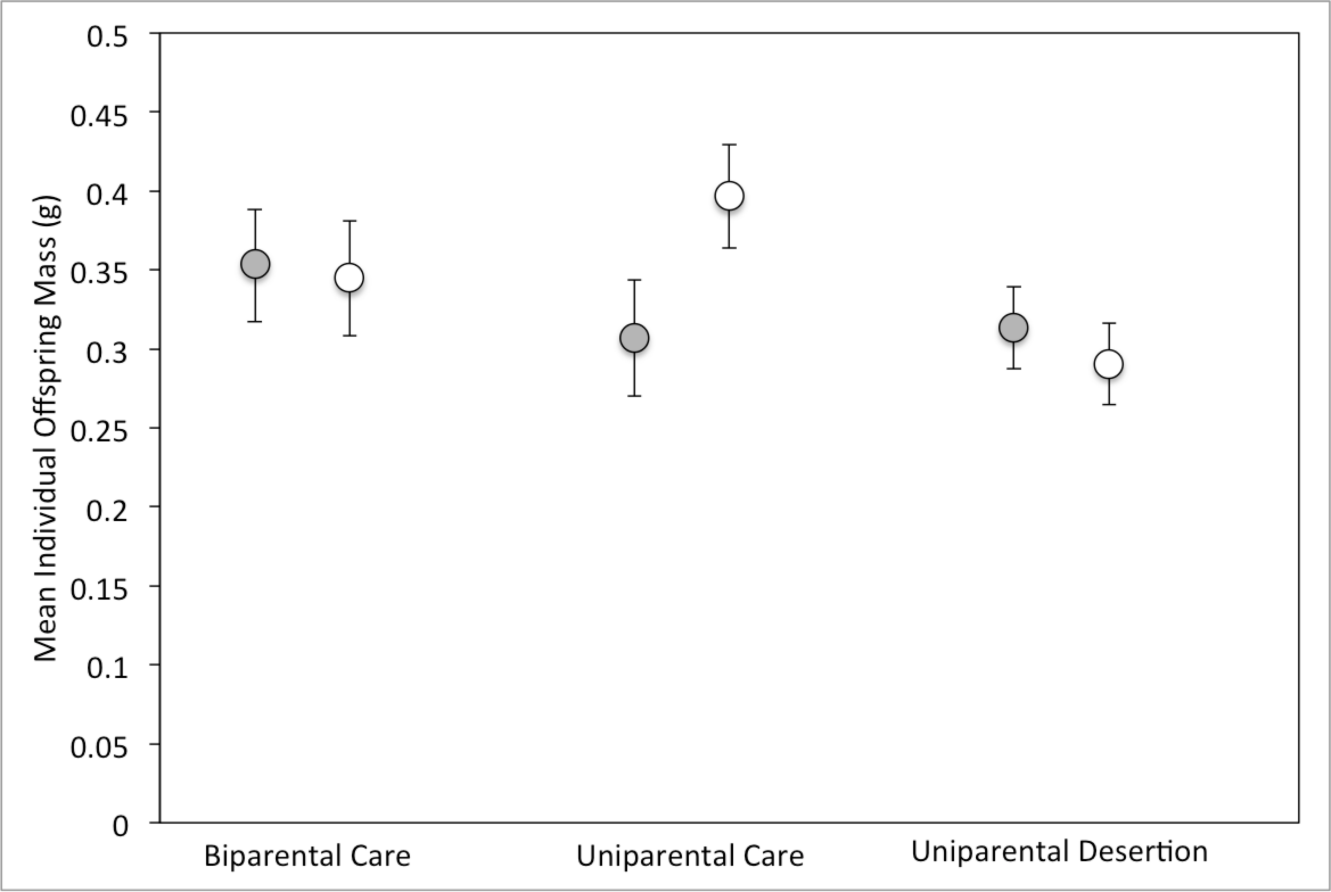
Mean (+/- 95% confidence interval) individual offspring mass for males (gray circles) and females (white circles) in all three experimental treatments.

Mean individual offspring mass differed significantly among parental types, and the interaction between parental type and sex was also significant (Table 1). Deserting females had lighter offspring than both biparental (p = 0.0186) and uniparental (p < 0.0001) females, but individual offspring mass did not differ between males of any parental type. In general, uniparental females have heavier individual offspring than males and females in all other treatments (Fig 3).

**Fig 4:**
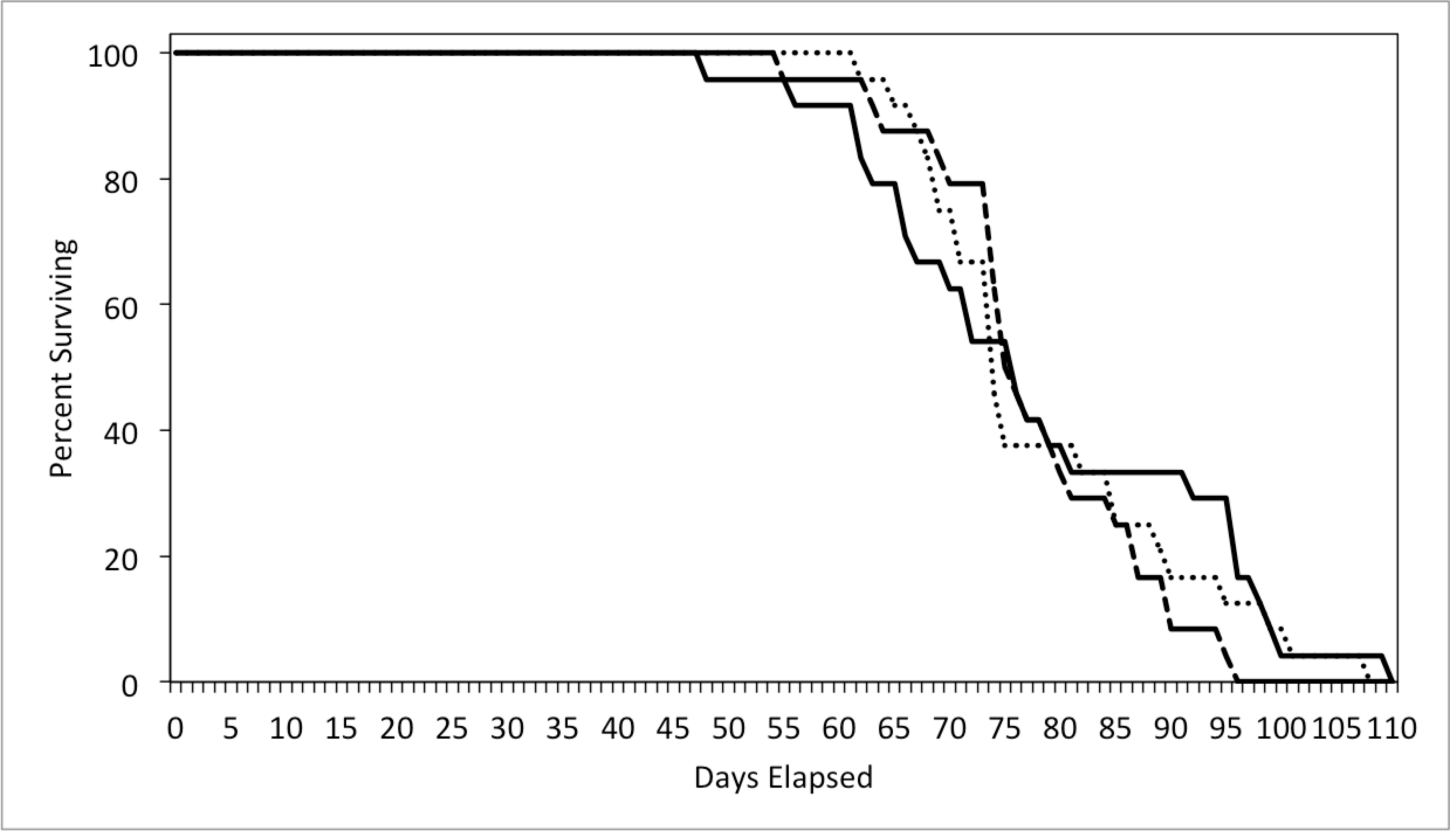
Survival curve for males and females combined in all three experimental treatments. Treatments are represented as: uniparental care–dotted line, biparental care–solid line, uniparental desertion–dashed line.

Lifespan differed significantly among parental types, but sex and the interaction between parental type and sex were not significant (Table 1). Uniparental care resulted in significantly longer lifespans of parents than biparental care (p = 0.0148) and desertion (p = 0.0156) strategies. Biparental care and desertion strategies resulted in shorter lifespans than uniparental care by about 8 days (Fig 4).

**Fig 5:**
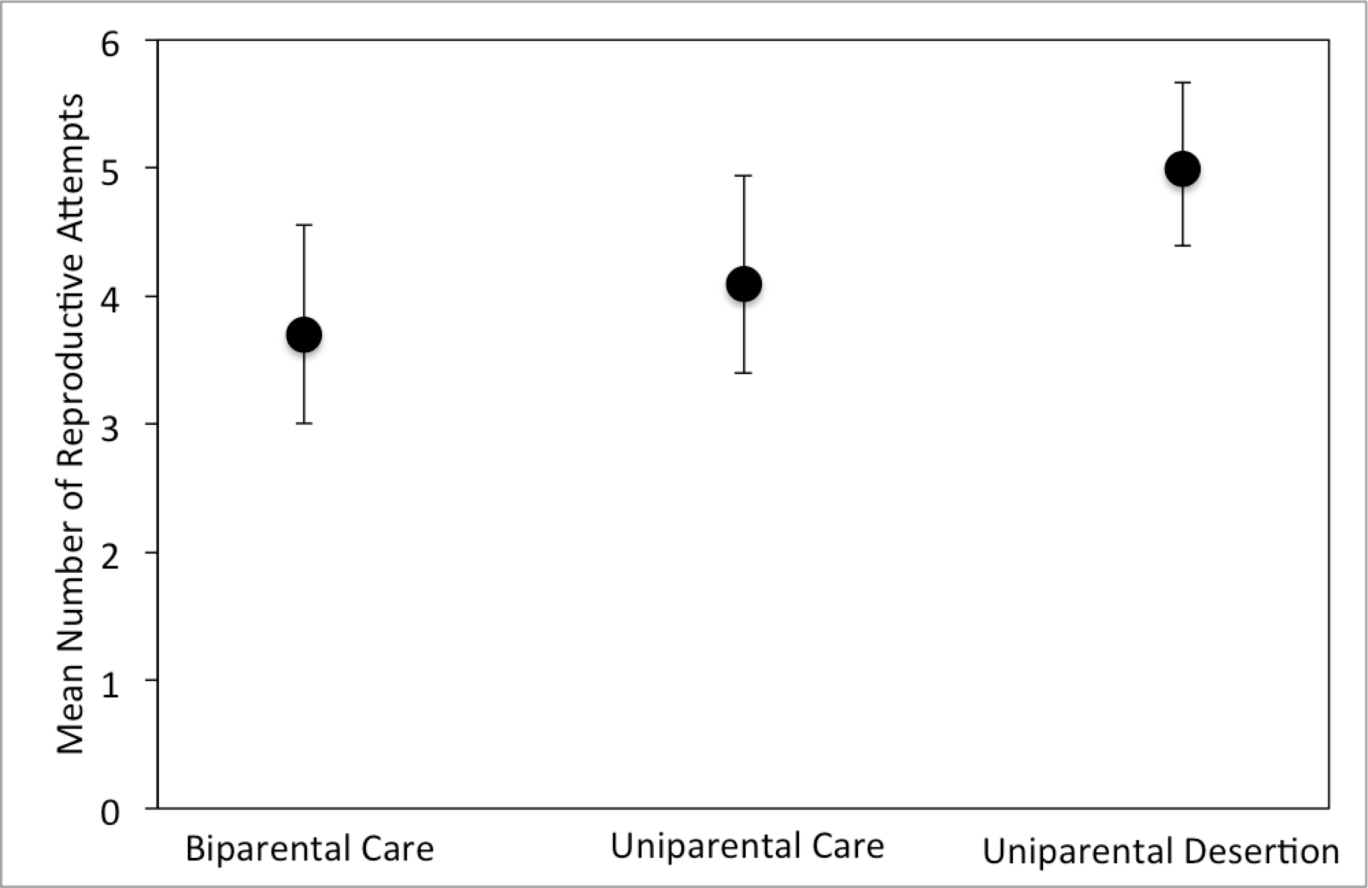
Mean (+/- 95% confidence interval) number of reproductive bouts by males and females combined in all three experimental treatments.

Total number of reproductive bouts differed significantly among parental types, but sex and the interaction between parental type and sex were not significant (Table 2). Biparental and uniparental care of offspring resulted in about one less reproductive bout over the lifetime compared to the desertion strategy (Fig 5).

**Table 2:** Analysis of variance (ANOVA) table for number of reproductive bouts.

Response Variable	Source	Num df/ Den df	Chi-Square value	p-value
Number of Reproductive Bouts	Sex	1/99	0.72	0.3954
	Parental Type	2/99	6.93	**0.0313**
	Parental Type*Sex	2/99	5.28	0.0715

Significant values are shown in bold.

**Fig 6:**
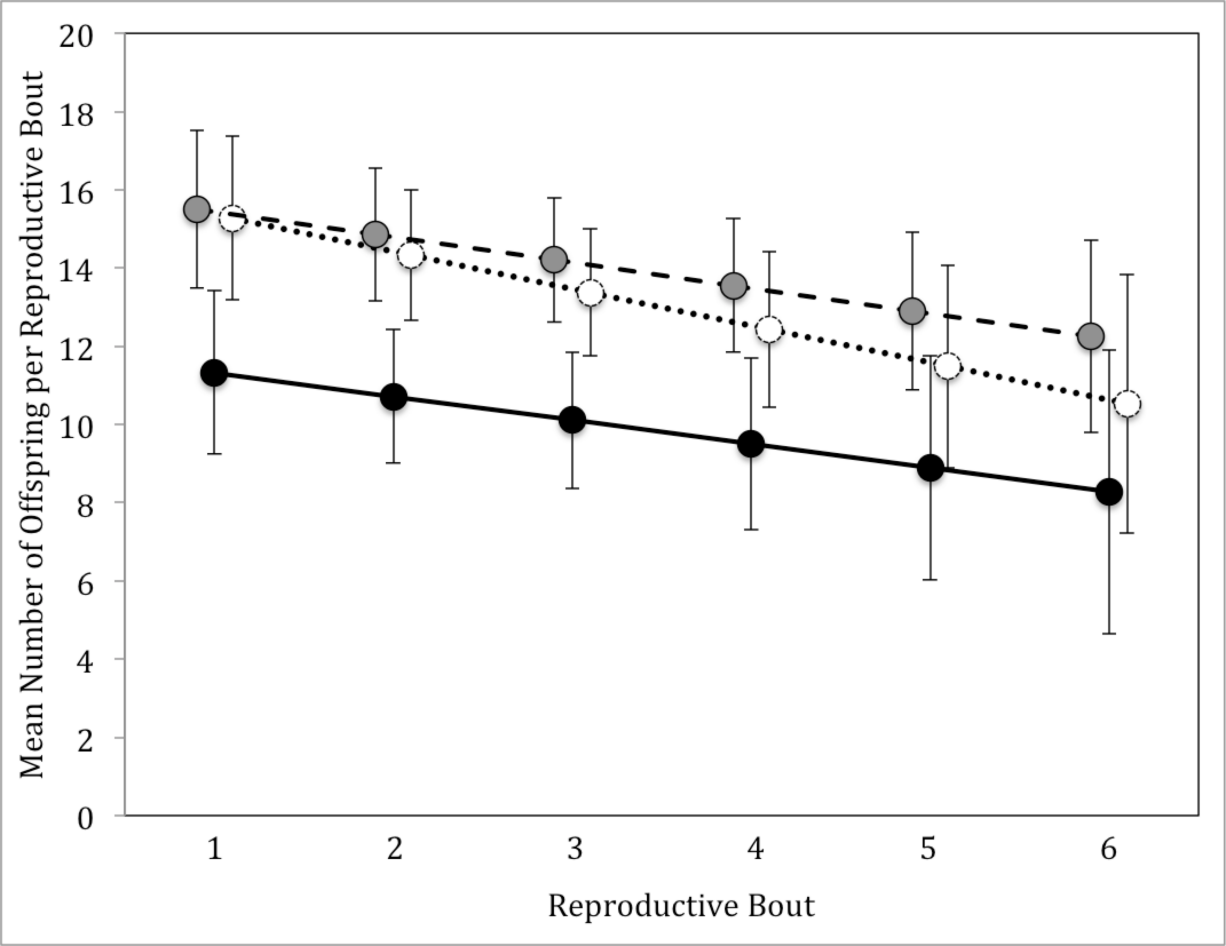
Mean (+/- standard deviation) number of offspring per reproductive bout for males and females combined in all three experimental treatments. Treatments are represented as: uniparental care–white circles with dotted line, biparental care–black circles with solid line, uniparental desertion–gray circles with dashed line.

Number of offspring per reproductive bout differed significantly among successive reproductive bouts and parental type had a significant effect, but sex and all possible interactions among sex, parental type, and reproductive bout were not significant (Table 3). Biparental care parents had about 4 fewer offspring per reproductive bout than both uniparental care and uniparental desertion treatments (Fig 6). In general, number of offspring per reproductive bout decreased with each successive attempt.

**Table 3:** Analysis of variance (ANOVA) table for number of offspring per reproductive bout.

Source	Num df/ Den df	F-value	p-value
Sex	1/252.1	3.64	0.0525
Parental Type	2/251.6	3.20	**0.0395**
Reproductive Bout	1/246.7	9.35	**0.0020**
Sex*Parental Type	2/251.6	1.36	0.2676
Reproductive Bout*Sex	1/246.7	1.38	0.2229
Reproductive Bout*Parental Type	2/245.4	0.21	0.8252
Reproductive Bout*Sex*Parental Type	2/245.4	0.48	0.6458

Significant values are shown in bold.

## Discussion

The first goal of this study was to test for a mutual fitness benefit of biparental care over the lifetime of *N*. *orbicollis* parents. We found that biparental care resulted in lower fitness for both sexes compared to both uniparental care and uniparental desertion strategies. Lifespan, lifetime number of offspring, total offspring mass produced, and number of offspring per reproductive bout were all lower under biparental care compared to the other conditions and number of reproductive bouts was lower than the uniparental desertion strategy. These results are inconsistent with the mutual benefit hypothesis for the evolution of biparental care. However, biparental care cannot evolve unless fitness obtained through biparental care is higher than that obtained through uniparental care or desertion [16].

If biparental care does not result in a greater number of offspring for either parent, how did biparental care evolve in this group? Male parental care could have evolved as a defense against predators and other male burying beetles (i.e. sexually-selected infanticide). Paternal care has evolved in groups where male parental care is important for offspring defense, such as in African lions [17] and langur monkeys [18]. In burying beetles, male presence may reduce the chances of a takeover by an intruder [5, 8, 19]. Under semi-natural conditions, male *N*. *orbicollis* intruders were successful at establishing residency on a carcass with a single female 25%–29% [8] and 12%-41% [5] of the time. However, when a pair of beetles was present, intruders established residency on a carcass only 0%-7% [8] and 6%-18% [5] of the time, depending on carcass size. These rates likely also vary due to differences in beetle density. Although our experimental design did not allow for take-over attempts by intruders, we demonstrate that *N*. *orbicollis* that exhibit biparental care experience a 33% decline in lifetime number of offspring compared to uniparental care or not providing parental care. Therefore, for biparental care to evolve, the effect of intruding beetles would have to result in a complete loss of the brood, at the minimum, on more than 33% of carcasses for uniparental or abandoning individuals. Thus, brood defense seems to be a potential explanation for the evolution of biparental care in burying beetles because pairs of parents are better able to defend a carcass from intruders than are single females [5, 8].

It is also possible that biparentally caring parents might trade off offspring number for offspring size to achieve the same fitness as uniparental parents. In burying beetles, body size is the main determinant of success in competitions for carcasses [20–25], and parents are able to produce larger offspring if they cull more of the brood [26]. However, in our study biparental care did not result in larger offspring (Fig 3), so it seems unlikely that biparentally caring parents produce fewer, larger offspring as an alternative strategy to increase their fitness. Instead, parents seem to use the same reproductive strategy regardless of whether they are caring for offspring alone or with a partner (Figs 1–3).

Male care may have evolved to increase paternity in the brood of the resident male [27]. For example, male African assassin bugs fight to guard a female’s egg mass, which likely ensures paternity [28]. In burying beetles, females mate multiply [29], which in *N*. *vespilloides*, results in approximately 15% of the offspring being sired by non-resident males [30]. Selection would favor males that remain with the brood if their presence and culling behavior increased their paternity [27]. Supporting this hypothesis, resident male *N*. *vespilloides* stayed with their partners longer and mated with them more frequently when other males were present, although the resident male’s presence did not affect reproductive output [31]. However, this does not fully explain why males remain with the brood after the offspring hatch. Post-hatching presence of females does increase maternity. In *N*. *vespilloides*, resident females tend to cull the first arriving offspring on the carcass, which are more likely to be from female brood parasites. As a result, maternity is skewed in the resident female’s favor [32–33]. Males may use a similar timing rule to cull offspring that belong to other males. By culling non-related offspring, males and females would be increasing the amount of food for their own offspring. However, this hypothesis is as yet untested.

Sexual conflict over parental investment is a strong selective force within the genus *Nicrophorus* [34–36]. In the current study, we demonstrate that the presence of one sex results in lower lifetime fitness for the other, creating conflict over presence of both sexes. Bonocoraglio and Kilner [12] found that *N*. *vespilloides* females benefited by not having males present, possibly because females are able to feed more on the carcass. However, their conclusion is confounded by their experimental design, which did not allow for natural dispersal from the carcass. Our study, which did allow for natural dispersal of post-reproductive parents, also demonstrated considerable costs to biparental parents. These costs included reduced lifespan, lifetime number of offspring, total offspring mass, individual offspring mass, and number of reproductive attempts.

The cost of parental care is expected to be a reduction in future survival or fecundity for the parent [2, 37]. Interestingly, not providing post-hatching parental care does not lead to increased fitness as measured by number or mass of offspring for either sex. We consider two possible explanations for this result. First, parental care by only the male may reduce the fitness of the female. Males tend to raise fewer offspring than do females on the same size of carcass [27], and the mass of individual offspring raised by uniparental males is lower than the mass of offspring raised by uniparental females (Fig 3). Therefore, females may not benefit from abandoning their offspring to be cared for by the male. Even though she may gain additional reproductive attempts, her fitness is dependent on male parental care behaviors, and thus she will realize lower fitness. Second, the cost of post-hatching parental care may be low relative to the benefit of readily available food. In *N*. *vespilloides*, females lived longer when their partners deserted the brood before the larvae provisioning stage, which may occur because females can feed more from the carcass in the absence of males [12]. Additionally, subordinate female *N*. *vespilloides* that were prevented from eating from the carcass produced fewer eggs than dominant females with access to the carcass [38]. Thus, the post-hatching period of parental care may be important for parents to feed from the carcass to store energy for the next reproductive bout and forcing a parent to abandon the brood and foregoing feeding from the carcass may reduce lifespan and/or future reproductive output.

This experiment offers a unique opportunity to address the costs of reproduction among uniparental, biparental, and desertion reproductive strategies. The evolution of biparental care is a complex process [39], and rigorous experimentation is necessary to determine the drivers of the evolution of this complex type of care. Our results indicate that biparental care is more costly than uniparental care for both males and females in terms of lifespan, and lifetime number and mass of offspring. However, loss of the brood to intruders may be common, all offspring may not be related to the resident pair, and resources for reproduction (and thus potential mates) may be limited under natural conditions. Thus, parental defense of offspring and parentage may select for evolution of biparental care in burying beetles. These potential drivers are not mutually exclusive, and a combination of these factors may have led to the evolution of parental care strategies in burying beetles.
